# Chloroform in Intermittent Fever

**Published:** 1869-03

**Authors:** D. Scott

**Affiliations:** Bellefontaine, Iowa


ARTICLE XII.

### CHLOROFORM IN INTERMITTENT FEVER.

BY D. SCOTT, M.D., Bellefontaine, Iowa.

N. S. DAVIS, M.D.: *Dear Sir:*—I beg leave to add a little more testimony on the use of chloroform as an internal remedy.

To Dr. A. P. Merrill, of New York, is due the credit of the discovery of this property of chloroform; although the medical journals, in their notice of my report on its use in the chill of intermittent fever, gave the credit to Dr. McClellan, who, certainly, has no more claim to originality in the use of the remedy than myself. I regard its discovery as a matter of no mean importance; on the contrary, I believe it to be second only to its original discovery as an anæsthetic. In support of Dr. Merrill's claims to priority in its use, I would beg leave to refer you to his published Lectures on Fever, delivered in the Memphis Medical College, in 1853–6, from which I make the following quotation, page 58:—"Chloroform, in doses of a fluid drachm, more or less, and repeated as its effects subside, will accomplish, in most cases, all that can be expected of opium, in regard to the nervous system, and without the objectionable features of the opium practice. * * * I have known the chill of an intermittent to be relieved by it more promptly and effectually than by any other remedy; moderating, also, the stage of reaction to an extent which rendered it almost imperceptible; thus placing the patient, with little loss of time, in a condition to receive proper treatment for the period of intermission. * * * No risk of fatal or injurious effects is incurred by the use of chloroform in drachm doses; but it must be borne in mind, that its effects are much less permanent and lasting than those of opium; a frequent repetition of the dose being necessary to a prolonged influence of the remedy." It is also mentioned on pages 81, 136, 169, 191, 193, 209, 230. It was not until after the perusal of the Doctor's report on the use of chloroform as an internal remedy, written for the International Medical Congress, that I became aware that the range of its application was so extended; since which time, I have used it in a variety of pathological conditions, such as infantile convulsions, puerperal convulsions, uterine hemorrhage (*post partum*), spasm of the stomach, colic, etc.

The following cases I desire to report in detail:—

Case I. On the 8th of July, 1868, I was hastily summoned to see a lady, 44 years of age, whom, six hours previously, I had delivered of a dead child, at full term: the case having been one of placenta prævia, I had turned and delivered, with little trouble to myself or patient, and left her, two hours afterwards, apparently comfortable and cheerful—but the messenger said she was dying; and, when I reached her, a few minutes later, I feared it was but too true. She was just emerging from a convulsion—the twelfth, the attendants informed me—there she lay, cold and cadaverous, bloody foam issuing from the mouth, respiration stertorous, and no pulse perceptible at the wrist; the jaws were spasmodically closed; I opened them, however, with some difficulty; and, in this dire extremity, I unhesitatingly poured half an ounce of undiluted chloroform into her mouth; which was immediately swallowed. In ten minutes the stertorous breathing ceased, the pulse came up full and bounding, and the patient was sleeping as easily and naturally as though enjoying the most healthful slumber. This continued for a period of about two hours, when she awoke perfectly conscious; but, with some confusion of mind, she made a slow but good recovery.

That the recovery, in this case, was due to the remedy, we may reasonably conclude, when we take into consideration the exhausted condition of the patient from flooding, etc., together with the severity and persistence of the paroxysms. Whether the remedy had ever been used previously in such condition, I am not advised; but, so favorably am I impressed with its efficacy, that whenever I am so unfortunate as to meet with such cases, I shall not hesitate to use it in heroic doses.

Case II. On the 20th August, 1868, I was hastily summoned to see a young woman who had ignorantly swallowed a quantity of strychnine, which quantity I could not exactly ascertain. I found her in great distress, having suffered repeated convulsions. I administered a teaspoonful of chloroform, which afforded considerable relief; but, no tendency to sleep, in fifteen minutes I repeated the dose; and, in a short time, she was sleeping very comfortably, and continued so for about one hour: she had no return of the symptoms, and no subsequent treatment.

Doubtless, the latter case develops nothing new, from the fact that Dr. Dresbach, of Tiffin, Ohio, reported a case of recovery, under similar treatment, a number of years ago.

